# Supplementary material for: The Firre locus produces a trans-acting RNA molecule that functions in hematopoiesis
Source: Nat Commun. 2019 Nov 13;10:5137. doi: 10.1038/s41467-019-12970-4 (PMC6853988; doi:10.1038/s41467-019-12970-4)
Supplement: Supplementary file 1 — Supplementary Information [file 41467_2019_12970_MOESM1_ESM.pdf]

Supplementary Information for:

Lewandowski et al., The *Firre* locus produces a trans-acting RNA molecule that functions in hematopoiesis

a

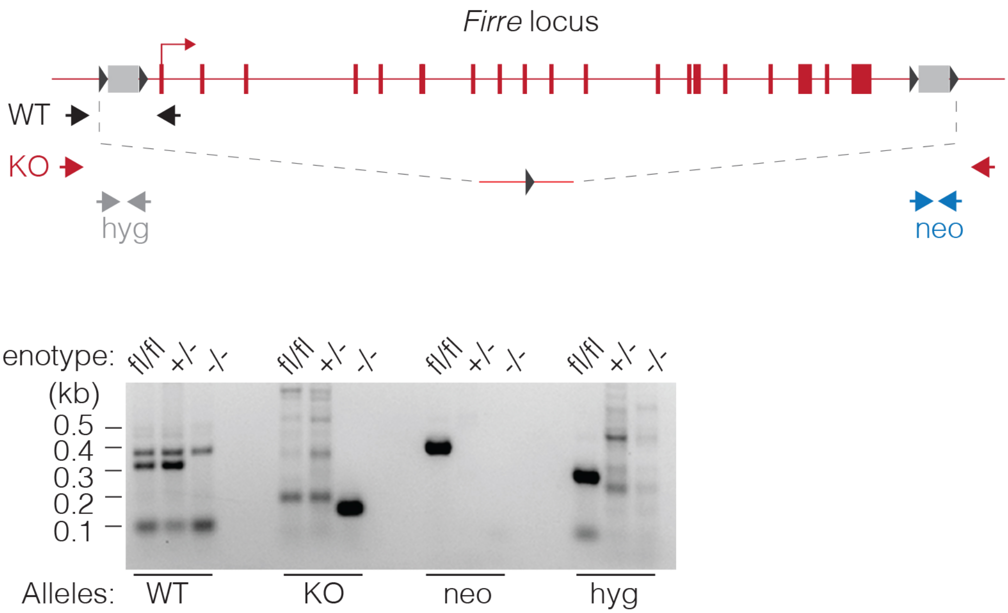

c

cggagctctagggtccttttatgtagcaaagtcaggcctgggaacagaacttcatg
tgcttgtaaaaagtctcaggtgcgtcctcgttgttttagagacttggagaggaatg
ggggcgggggaacaaaatccgaggacagtcgagccaagaaaagtcggggcttcta
ggatgccaaccacgccaaacagatcaaaaccaggactggaggactgaagatgaagc
cggcaaaaagtcaccagccacggctcttgtaaggtatgcttcacctctcctgctaa
gtcttcatccctgtctatgaggacaaagaagttacgggttaaattggcgatgggcc
aggcgctccttgaggatgctctaaagttggtgatagaaaatgggagaactgaagac
aaccactttataaaacccttgcctgcttgaatttgt

Supplementary Figure 1: Schematization of the targeted *Firre* locus and genotyping. (A) Targeted Firre locus shown in reverse orientation. Targeting cassettes containing hygromycin and neomycin cassettes shown as light gray rectangles and the loxP sites shown as dark gray triangles. Cre-mediated recombined allele shown below as a red line with a single loxP site. Arrows indicate genotyping primers used to amplify alleles for: Firre WT, black; knockout allele (KO), red; hygromycin (hyg), light gray; and neomycin (neo), blue. **(B)** Genotyping gel for: Firre^{floxed} (fl/fl); Firre heterozygous (+/-); and Firre knockout (-/-) mice. Alleles amplified indicated below the gel. **(C)** DNA sequence used to generate a Firre riboprobe.

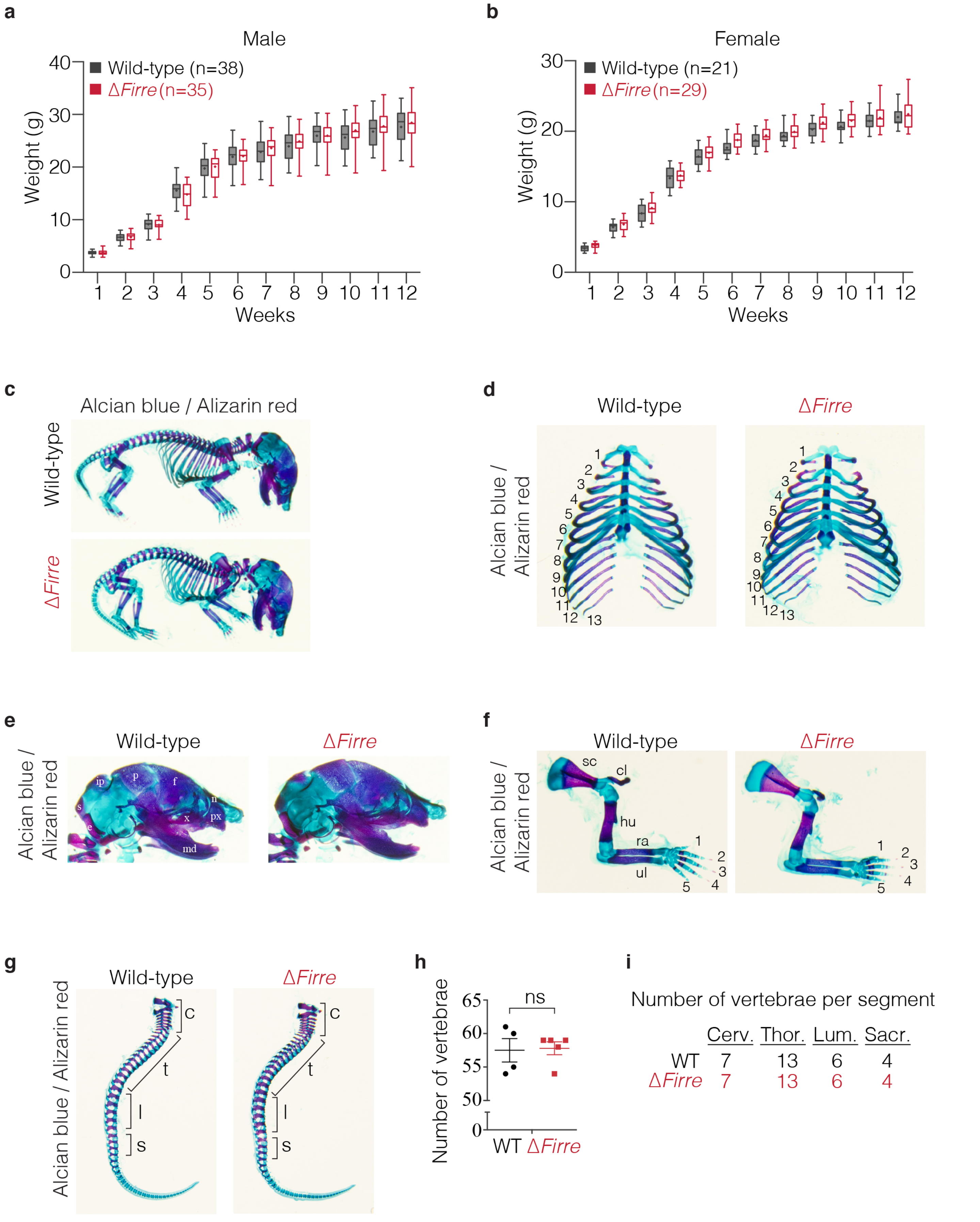

Supplementary Figure 2. Weight measurements and skeletal analysis of ΔFirre mice. (A) Body weight measurements in grams (g) for male WT (n=38) (gray) and ΔFirre (n=35) (red), and **(B)** female WT (n=21) (gray) and ΔFirre (n=29) (red) mice over 12 weeks. Data shown as a box and whisker plot with error bars showing the minimum and maximum, the significance was determined using a two-tailed t-test. **(C-G)** Skeletal preparations of E18.5 WT (n=8) and ΔFirre (n=7) mice stained with alcian blue (cartilage) and alizarin red (bone) show that ΔFirre mice appear to have normal skeletal development. **(D)** Rib cages from E18.5 wild-type (n=8) ΔFirre (n=7) showing that ΔFirre embryos have a normal number of ribs. **(E)** Skulls from E18.5 WT (n=8) and ΔFirre (n=7) embryos show normal morphology. Abbreviations used: n, nasal; f, frontal bone; p, parietal; ip, interparietal; s, supraoccipital; e, exoccipital; md, mandible; and x, maxillary. **(F)** Limb patterning and ossification appears normal in WT (n=8) and ΔFirre mice (n=7). Abbreviations used: sc, scapula; cl, clavicle; hu, humerus; ra, radius; and ul, ulna. **(G)** Vertebrae patterning and ossification appears normal in WT (n=8) and ΔFirre (n=7) embryos. **(H)** The total number of vertebrae in E18.5 WT (n=4) and ΔFirre (n=5) embryos do not significantly differ (P=0.876, two-tailed unpaired t-test). Data plotted showing the mean ± SEM. **(I)** The number of vertebrae per: c, cervical; t, thoracic; l, lumbar, and s, sacral segments in E18.5 ΔFirre (n=5) embryos is the same as found in WT (n=4).

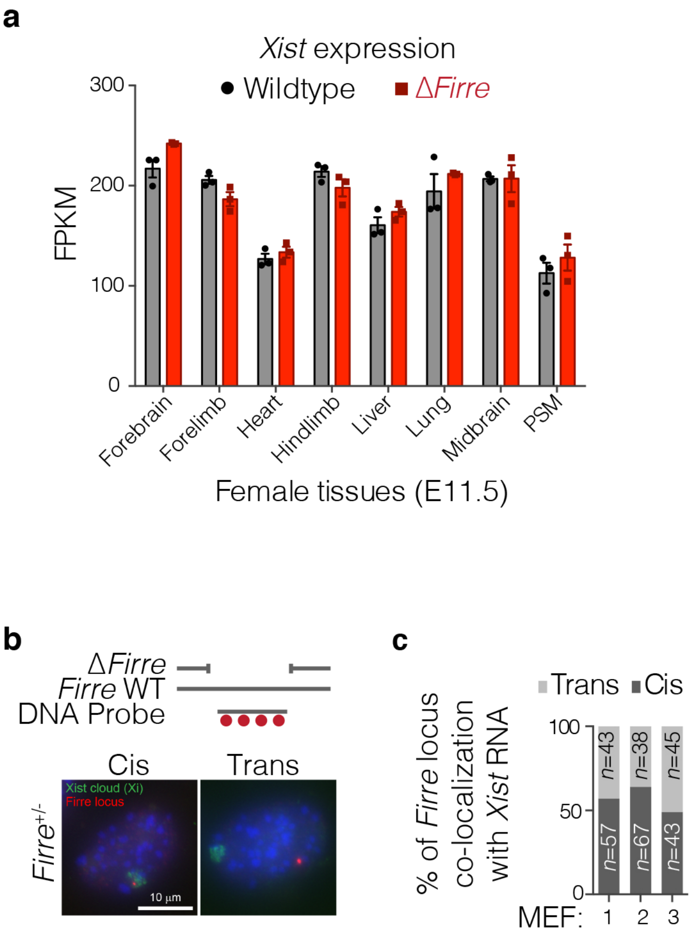

Supplementary Figure 3. Deletion of Firre does not impact X chromosome inactivation or change expression of Xist RNA. (A) Xist RNA expression (FPKM) in eight female tissues from RNA-seq in WT (n=3) and ΔFirre (n=3) at E11.5. Data are shown as mean ± SEM. **(B,C)** Co-DNA/RNA FISH in female Firre+/- MEFs. DNA FISH for the WT Firre locus shown in red and Xist RNA shown in green. Scale bar equals 10 micrometers. Quantification of localization of Xist RNA with the WT Firre locus for three independent Firre+/- MEFs. Cis indicates a co-localization between the WT Firre DNA locus and Xist RNA and trans indicates Xist RNA did not co-localize with the WT Firre DNA locus.

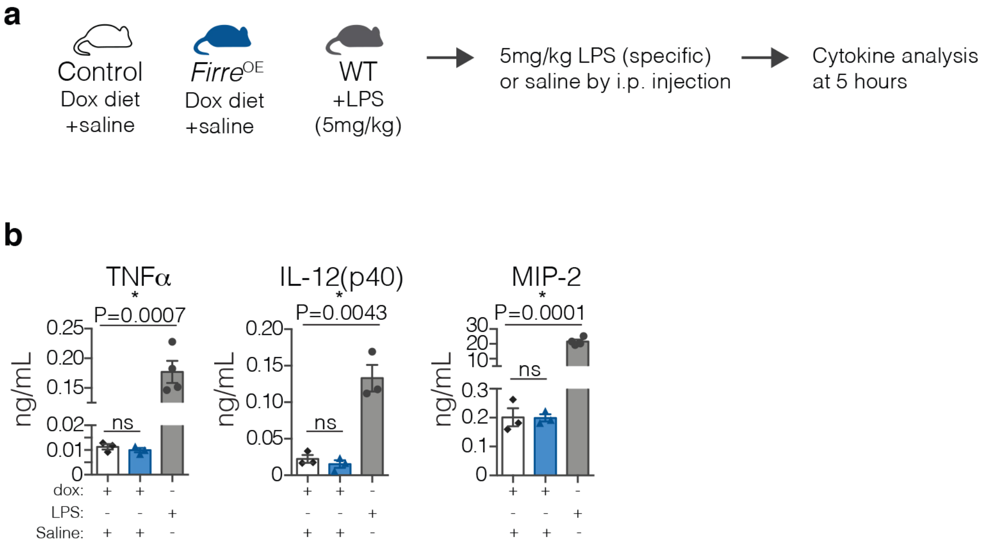

Supplementary Figure 4. Mice overexpressing Firre do not have increased serum cytokines. (A) Experimental schematic for cytokine measurements in 5-7 weeks old mice injected with either saline or LPS. **(B)** Cytokine measurements in serum at 5 hours post saline or LPS injection from control saline injected mice (WT or tg(Firre) fed a dox diet, n=3, black diamonds), Firre^{OE} saline injected mice fed a dox diet (n=3, blue triangles), and WT mice fed a normal diet injected with 5 mg/kg LPS (specific-activity) (n=3 to 4, gray circles). Data are shown as mean ± SEM and statistical significance determined using a two-tailed unpaired t-test.

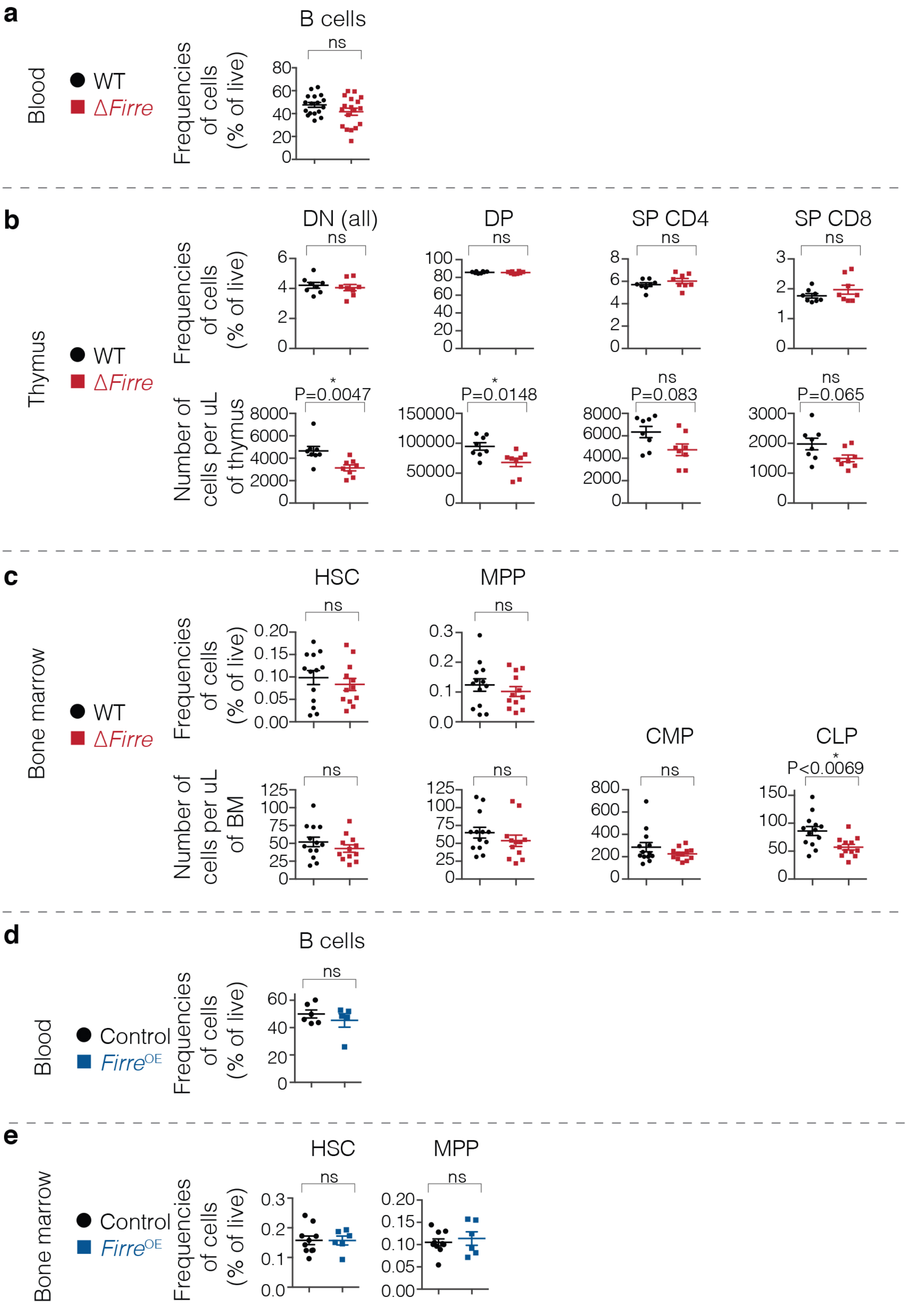

Supplementary Figure 5. Immunophenotyping in WT, ΔFirre, and Firre^{OE} mice. (A) Frequency of B cells in the peripheral blood shown as percent (%) of live cells from WT (n=17) and ΔFirre mice (n=18). Three representative experiments combined (seven independent experiments). **(B)** Frequencies of double negative (DN) (DN1, DN2, DN3, DN4), double positive (DP), single positive (SP) CD4, and SP CD8 cells in thymuses shown as percent of live cells from WT (n=8) and ΔFirre (n=8) mice. Enumeration of cells shown below as cells per µL of thymus. A representative experiment shown (three independent experiments). **(C)** Frequencies of HSC and MPP cell populations from total bone marrow (BM) shown as percent of live cells from WT (n=13) and ΔFirre (n=12) mice. Enumeration of cells shown below as cells per µL of bone marrow. Two representative experiments combined (three independent experiments). **(D)** Frequency of B cells in the peripheral blood shown as percent of live cells from control (tg(Firre), WT, or rtTA with dox) (n=6) and Firre^{OE} +dox (n=5) mice. One representative experiment shown (three independent experiments). **(E)** Frequencies of HSC and MPP cells from total BM shown as percent of live cells form control (tg(Firre), WT, or rtTA with dox) (n=10) and Firre^{OE} +dox mice (n=6) (two independent experiments). All data shown as mean ± SEM and statistical significance determined using a two-tailed Mann Whitney-U test.

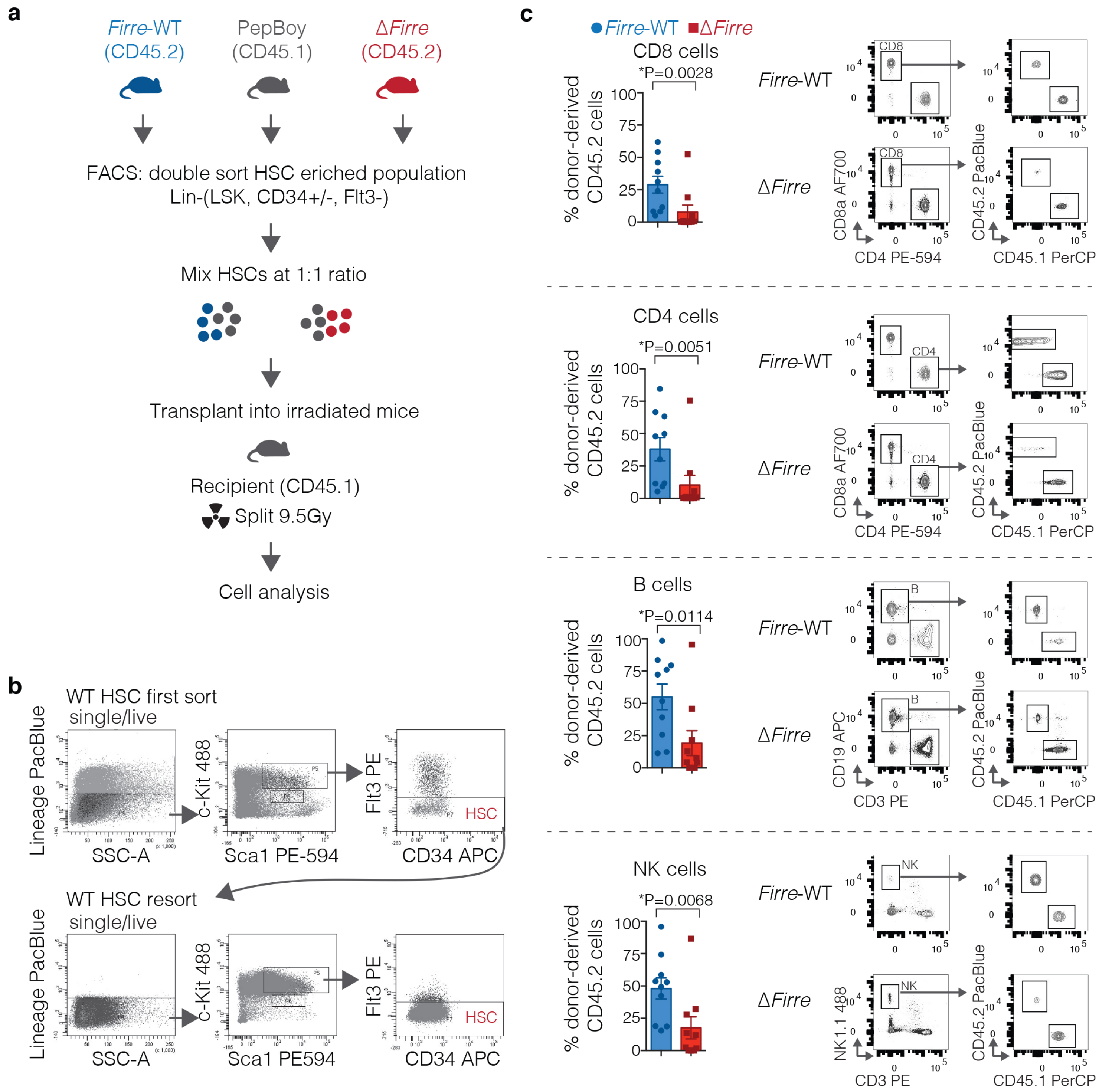

Supplementary Figure 6. ΔFirre HSCs are less competitive at repopulating blood in vivo. (A) Schematic of competitive chimera HSC transplant experiment. HSC enriched population from age- and sex-matched Firre WT/CD45.2 (blue) or ΔFirre/CD45.2 (red) combined with PepBoy/CD45.1 (gray) at a 1:1 ratio and transplanted into lethally irradiated PepBoy/CD45.1 recipient male mice. **(B)** Representative flow cytometry plots from WT showing the FACS strategy used for isolating an HSC-enriched population for transplant from lineage depleted total bone marrow. **(C)** Frequencies of donor-derived CD45.2 CD4, CD8, NK, and B cells at 23 weeks post competitive chimera transplant for Firre WT/CD45.2 with PepBoy/CD45.1 (n=10), and ΔFirre/CD45.2 with PepBoy/CD45.1 (n=10) (two independent experiments shown). Data are shown as mean ± SEM and significance determined by a two-tailed Mann-Whitney U test.

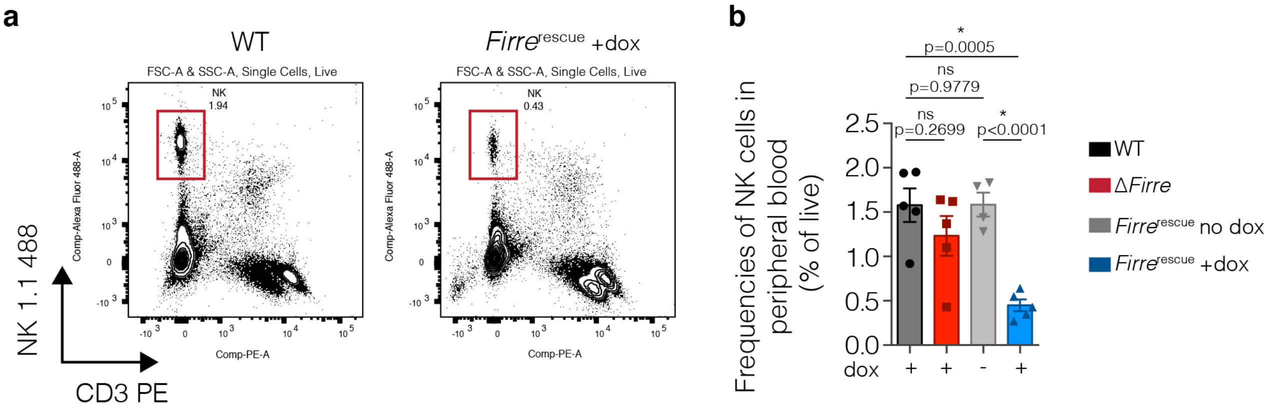

Supplementary Figure 7. Frequencies of NK cells in the blood in Firre transgenic mice. (A) Representative flow cytometry plots of NK cells in WT and Firre^{rescue} +dox mice. (B) Frequencies of NK cells shown as percent (%) of live cells in the peripheral blood from female mice 24 to 33 weeks old: dox-treated WT (n=5), dox-treated ΔFirre (n=5), no dox Firre^{rescue} (n=4), and dox-treated Firre^{rescue} (n=5). Data are shown as mean ± SEM, two independent experiments, and significance determined by using a two-tailed unpaired t-test.

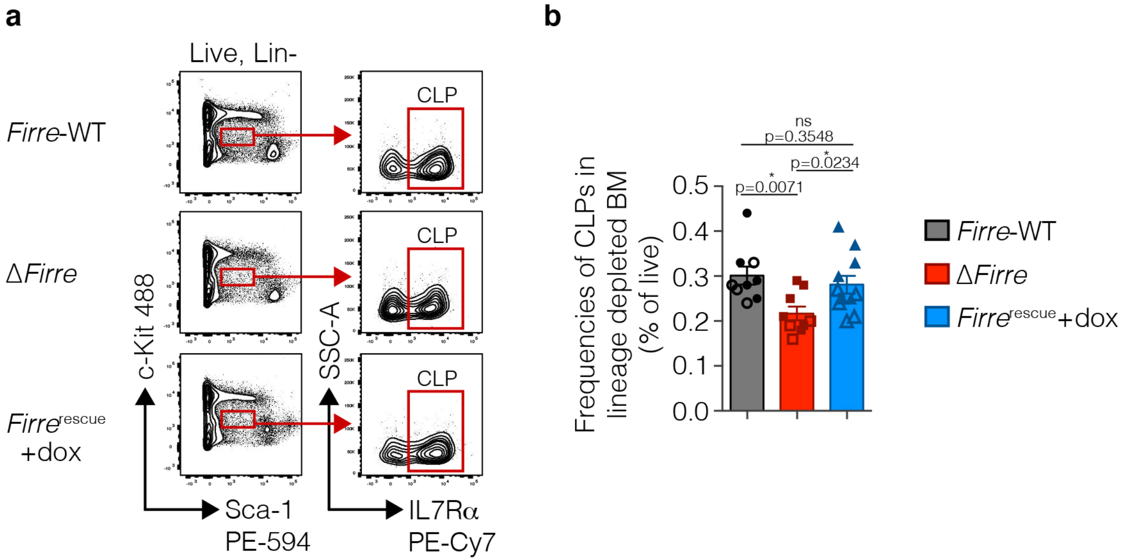

Supplementary Figure 8. Frequencies of CLPs in lineage-depleted bone marrow in Firre transgenic mice. **(A)** Representative gating strategy for identifying CLPs in lineage depleted bone marrow (BM) from WT, ΔFirre, and Firrerescue +dox mice. **(B)** Frequencies of CLPs shown as percent (%) of live cells in lineage depleted bone marrow over three experiments from male (7 to 10 weeks old, solid object) and female (19 to 24 weeks old, outlined object) mice: WT (n=9); ΔFirre (n=9), and dox-treated Firre^{rescue} (n=11). Data are plotted as the mean ± SEM and significance determined by a two-tailed Mann-Whitney U test.

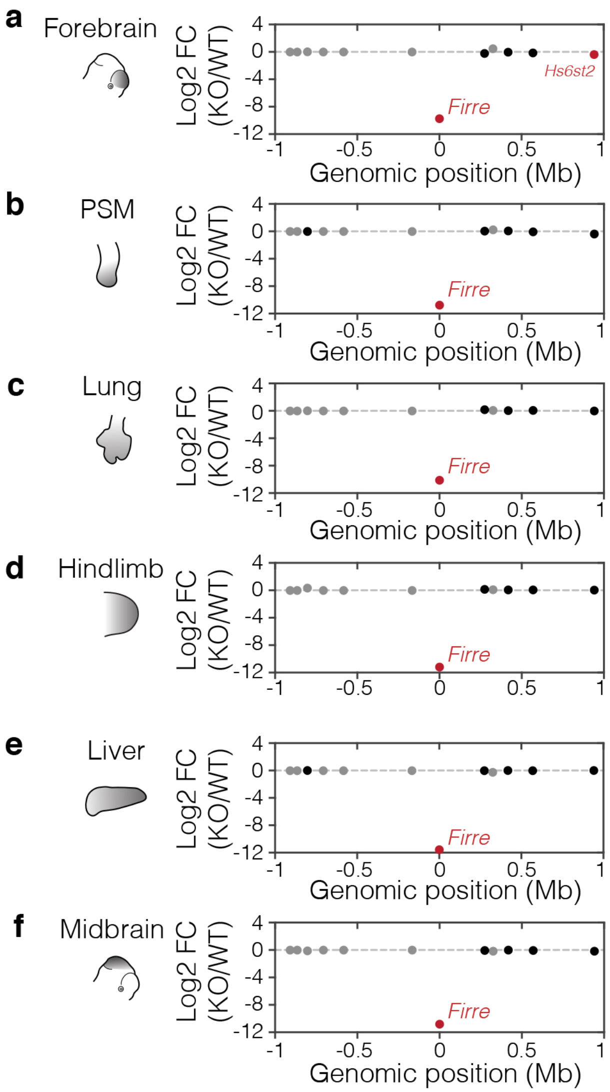

Supplementary Figure 9. Firre does not regulate the expression of neighboring genes. (A-F) Firre locus region (2 Mb) showing log2 fold change (log2 FC) gene expression differences (RNA-seq) between Δ*Firre* and WT E11.5 tissues (forebrain, pre-somitic mesoderm (PSM), lung, hindlimb, liver, and midbrain). Firre is shown in red, significantly dysregulated genes are shown in red, genes with less than 1 FPKM expression are shown in gray, and genes that are not significantly changed are shown in black.

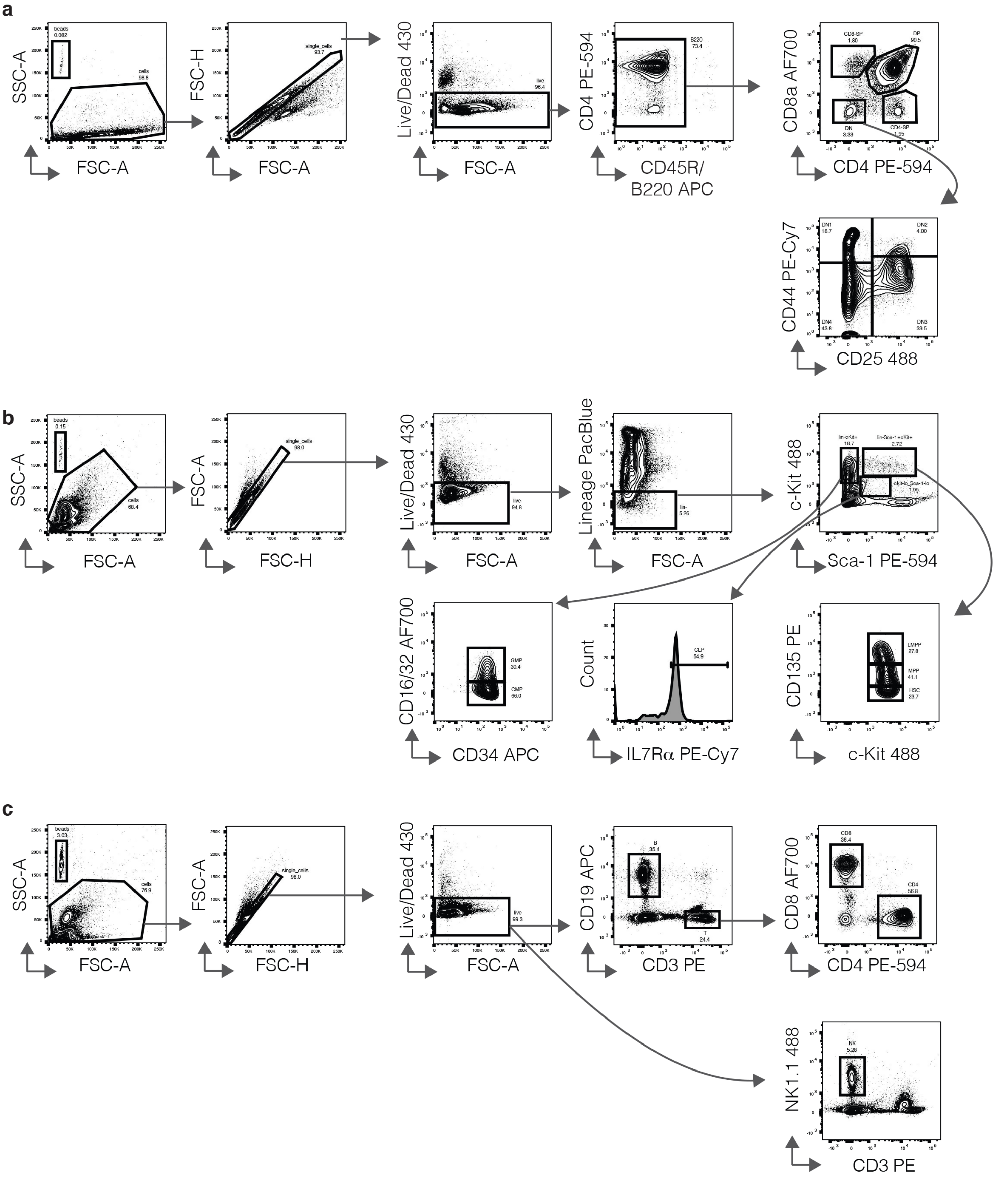

Supplementary Figure 10. Representative gating strategies for flow cytometry. (A) Gating strategy for thymocyte cell analysis: SP CD4 and CD8, DN 1-4, and DP CD4 and CD8. (B) Gating strategy in total bone marrow for CMP, CLP, HSC, and MPP cells. (C) Gating strategy in peripheral blood for B cell, NK, CD4, and CD8 cells.

Mating Genotype			Diet	Litters	Total pups	Mean litter size (±sd)	Progeny Genotype	Total number pups per genotype	♂	♀	n.d	*P* value
♂*Firre*$^{+/y}$	x	♀*Firre*$^{+/+}$	Normal	6	39	6.5±1.3	Wildtype	39	20	19	0	0.873
♂*Firre*$^{-/y}$	x	♀*Firre*$^{-/-}$	Normal	10	68	6.8±1.9	Δ*Firre*	68	30	38	0	0.332
♂rtTA	x	♀*Firre*^{OE}	Control	10	66	6.6±1.5	*Firre*^{OE}; *rtTA*	21	08	13	0	0.2752
							Firre^{OE}	23	11	07	05	
							rtTA	11	03	01	07	
							Wildtype	09	02	04	03	
							n.d	02				
♂rtTA	x	♀*Firre*^{OE}	Dox.	33	206	5.7±1.7	*Firre*^{OE}; *rtTA*	41	19	22	0	0.6394
							Firre^{OE}	76	35	28	13	
							rtTA	22	11	10	01	
							Wildtype	57	17	11	29	
							n.d	10				

Supplementary Table 1. Loss or gain of Firre expression does not alter sex distribtion. (A) Deletion of the Firre locus does not significantly alter the distribution of male and female progeny from matings between male and female ΔFirre mice, or in matings between male and female Firre WT mice (above dashed line). Matings between male rtTA and female Firre^{OE} mice on a dox or control diet do not show a significant difference in the distribution of male and female progeny that overexpress or do not overexpress Firre (Firre^{OE}; rtTA) (below dashed line). Tissue collection for genotyping performed at P7. Litter size shown as mean with standard deviation (s.d.), not determined (n.d.), Chi-square statistic reported (p-value).
