# Supplementary material for: The Firre locus produces a trans-acting RNA molecule that functions in hematopoiesis
Source: Nat Commun. 2019 Nov 13;10:5137. doi: 10.1038/s41467-019-12970-4 (PMC6853988; doi:10.1038/s41467-019-12970-4)
Supplement: Supplementary file 3 — Description of Additional Supplementary Files [file 41467_2019_12970_MOESM3_ESM.pdf]

Description of Additional Supplementary Files

File Name: Supplementary Data 1
Description: Differential gene expression in midbrain tissue from E11.5 wild-type and ΔFirre embryos.

File Name: Supplementary Data 2
Description: Differential gene expression in forebrain tissue from E11.5 wild-type and ΔFirre embryos.

File Name: Supplementary Data 3
Description: Differential gene expression in presomitic mesoderm tissue from E11.5 wild-type and ΔFirre embryos.

File Name: Supplementary Data 4
Description: Differential gene expression in lung tissue from E11.5 wild-type and ΔFirre embryos.

File Name: Supplementary Data 5
Description: Differential gene expression in hindlimb tissue from E11.5 wild-type and ΔFirre embryos.

File Name: Supplementary Data 6
Description: Differential gene expression in forelimb tissue from E11.5 wild-type and ΔFirre embryos.

File Name: Supplementary Data 7
Description: Differential gene expression in liver tissue from E11.5 wild-type and ΔFirre embryos.

File Name: Supplementary Data 8
Description: Differential gene expression in heart tissue from E11.5 wild-type and ΔFirre embryos.

File Name: Supplementary Data 9
Description: Differential gene expression in male CLPs from wild-type and ΔFirre.

File Name: Supplementary Data 10
Description: Differential gene expression in male CLPs from ΔFirre and ΔFirre; $Firre^{rescue}$.
