# Supplementary material for: The Firre locus produces a trans-acting RNA molecule that functions in hematopoiesis
Source: Nat Commun. 2019 Nov 13;10:5137. doi: 10.1038/s41467-019-12970-4 (PMC6853988; doi:10.1038/s41467-019-12970-4)
Supplement: Supplementary file 14 — Reporting Summary [file 41467_2019_12970_MOESM14_ESM.pdf]

nature research

Corresponding author(s): John Rinn

Last updated by author(s): 6-27-2019

Reporting Summary

Nature Research wishes to improve the reproducibility of the work that we publish. This form provides structure for consistency and transparency in reporting. For further information on Nature Research policies, see Authors & Referees and the Editorial Policy Checklist.

Statistics

For all statistical analyses, confirm that the following items are present in the figure legend, table legend, main text, or Methods section.

n/a	Confirmed	
☐	☒	The exact sample size (n) for each experimental group/condition, given as a discrete number and unit of measurement
☐	☒	A statement on whether measurements were taken from distinct samples or whether the same sample was measured repeatedly
☐	☒	The statistical test(s) used AND whether they are one- or two-sided<br>*Only common tests should be described solely by name; describe more complex techniques in the Methods section.*
☐	☒	A description of all covariates tested
☐	☒	A description of any assumptions or corrections, such as tests of normality and adjustment for multiple comparisons
☐	☒	A full description of the statistical parameters including central tendency (e.g. means) or other basic estimates (e.g. regression coefficient) AND variation (e.g. standard deviation) or associated estimates of uncertainty (e.g. confidence intervals)
☐	☒	For null hypothesis testing, the test statistic (e.g. F, t, r) with confidence intervals, effect sizes, degrees of freedom and P value noted<br>*Give P values as exact values whenever suitable.*
☒	☐	For Bayesian analysis, information on the choice of priors and Markov chain Monte Carlo settings
☒	☐	For hierarchical and complex designs, identification of the appropriate level for tests and full reporting of outcomes
☒	☐	Estimates of effect sizes (e.g. Cohen's d, Pearson's r), indicating how they were calculated

Our web collection on statistics for biologists contains articles on many of the points above.

Software and code

Policy information about availability of computer code

Data collection	FACS Diva Software (BD) was used to collect cells by fluorescence activated cell sorting.
Data analysis	FlowJo software 10.4.1 (Treestar) used for flow cytometry analysis. Prism 6.0h (GraphPad) was used to generate graphs and perform statistical analysis. Publicly available software: FeatureCounts v1.6.2, DESeq2 v1.14.1 , fdrtools v1.2.15, TopHat v2.1.1, Cufflinks v2.2.1 and Cuffdiff were used in RNA-sequencing analyses.

For manuscripts utilizing custom algorithms or software that are central to the research but not yet described in published literature, software must be made available to editors/reviewers. We strongly encourage code deposition in a community repository (e.g. GitHub). See the Nature Research guidelines for submitting code & software for further information.

Data

Policy information about availability of data

All manuscripts must include a data availability statement. This statement should provide the following information, where applicable:

- Accession codes, unique identifiers, or web links for publicly available datasets
- A list of figures that have associated raw data
- A description of any restrictions on data availability

RNA-sequencing data is available at Gene Expression Omnibus under the accession numbers GSE125683 and GSE83631. The data that support the findings of the study are available from the corresponding author upon request.

Field-specific reporting

Please select the one below that is the best fit for your research. If you are not sure, read the appropriate sections before making your selection.

☒ Life sciences ☐ Behavioural & social sciences ☐ Ecological, evolutionary & environmental sciences

For a reference copy of the document with all sections, see nature.com/documents/nr-reporting-summary-flat.pdf

Life sciences study design

All studies must disclose on these points even when the disclosure is negative.

Sample size	No statistical methods were used to predetermine sample size. For RNA-seq experiments, the sample size was determined based on what is currently accepted in the literature (n>3 per group). For in vivo animal experiments, we estimated the sample size based on our pilot experiments, previous work, and by careful assessment of the literature.
Data exclusions	Samples with technical failure during processing for cytokine assessment or flow cytometry were excluded from analyses.
Replication	Data are representative of multiple independent experiments.
Randomization	Mice of different genotypes were assigned a number at the tissue collection date and were identified as the number throughout data collection and data analysis for for experiments involving cell analysis by flow cytometry and cytokine analysis.
Blinding	At the tissue collection stage for experiments involving cell analysis by flow cytometry and cytokine analysis, investigators referenced individual mice by assigned number.

Reporting for specific materials, systems and methods

We require information from authors about some types of materials, experimental systems and methods used in many studies. Here, indicate whether each material, system or method listed is relevant to your study. If you are not sure if a list item applies to your research, read the appropriate section before selecting a response.

Materials & experimental systems

n/a	Involved in the study	
☐	☒	Antibodies
☒	☐	Eukaryotic cell lines
☒	☐	Palaeontology
☐	☒	Animals and other organisms
☒	☐	Human research participants
☒	☐	Clinical data

Methods

n/a	Involved in the study	
☒	☐	ChIP-seq
☐	☒	Flow cytometry
☒	☐	MRI-based neuroimaging

Antibodies

Antibodies used	The antibodies used in this study are also listed and described in the methods under the section titled Flow Cytometry Analysis. Alexa Fluor 700 anti-mouse CD8a clone 53-6.7 (Biolegend, 100730), PE/Dazzle-594 anti-mouse CD4 clone GK1.5 (Biolegend, 100456), APC anti-mouse CD19 clone 6D5 (Biolegend, 115512), Alexa Fluor 488 anti-mouse NK-1.1 clone PK136 (Biolegend, 108718), PE anti-mouse CD3 clone 17A2 (Biolegend, 100205), TruStain FcX (anti-mouse CD16/32) clone 93 (1:50) (Biolegend, 101319), Pacific Blue anti-mouse CD45.2 clone 104 (Biolegend, 109820), PerCP anti-mouse CD45.1 clone A20 (Biolegend, 110726), Fluor 488 anti-mouse CD25 clone PC61 (Biolegend, 102017), PE/Cy7 anti-mouse/human CD44 clone IM7 (Biolegend, 103030), PE anti-mouse TCR β chain clone H57-597 (Biolegend, 109208), APC anti-mouse/human CD45R/B220 clone RA3-6B2 (Biolegend, 103212), eFluor 450 anti-Mouse CD69 clone H1.2F3 (Invitrogen/eBioscience, 48069182), Alexa Fluor 700 anti-Mouse CD16/CD32 clone 93 (Invitrogen/eBioscience, 56-0161-82), PE/Cy7 anti-mouse CD127 (IL-7Rα) clone A7R34 (Biolegend, 135014), Alexa Fluor 488 anti-mouse CD117 (c-Kit) clone 2B8 (Biolegend, 105816), PE/Dazzle-594 anti-mouse Ly-6A/E (Sca-1) clone D7 (Biolegend, 108138), APC anti-mouse CD34 clone HM34 (Biolegend, 128612), PE anti-mouse CD135 clone A2F10 (Biolegend, 135306), and Pacific Blue anti-mouse Lineage Cocktail clones 17A2/RB6-8C5/RA3-6B2/Ter-119/M1/70 (Biolegend, 133310)
Validation	Antibodies specific for the required antigens / epitopes were purchased from commercial vendors. Specific clones were chosen based on use in the literature, our own pilot studies, and by the product data sheets.

Animals and other organisms

Policy information about studies involving animals; ARRIVE guidelines recommended for reporting animal research

Laboratory animals	Mouse strains for experiments in this study are described in Methods under the section titled Firre Mouse Strains and

Laboratory animals	Genotyping, and information is also indicated in the figure legends. Mouse strains used in this study: C57BL6/J (JAX); B6.C-Tg(CMV-Cre)1Cgn/J (Jackson Lab, 006054); Firrefloxed (029931, Jackson Lab); ΔFirre (030038, Jackson Lab); tg(Firre) (C57BL6/J); PepBoy/CD45.1 (Jackson Laboratory, 002014)
Wild animals	Not applicable
Field-collected samples	Not applicable
Ethics oversight	The Institutional Animal Care and Use Committee (IACUC) is the standing committee on animals in research at Harvard University.

Note that full information on the approval of the study protocol must also be provided in the manuscript.

Flow Cytometry

Plots

Confirm that:

☒ The axis labels state the marker and fluorochrome used (e.g. CD4-FITC).

☒ The axis scales are clearly visible. Include numbers along axes only for bottom left plot of group (a 'group' is an analysis of identical markers).

☒ All plots are contour plots with outliers or pseudocolor plots.

☒ A numerical value for number of cells or percentage (with statistics) is provided.

Methodology

Sample preparation	Cells were isolated from the peripheral blood, bone marrow, and thymus and prepared as described in the Methods under the section titled Flow Cytometry Analysis.
Instrument	LSR-II (BD Biosciences) and Aria II (BD Biosciences)
Software	FACS Diva software (BD) was used during FACS and FlowJo software 10.4.1 (Treestar) was used for cell analysis.
Cell population abundance	*Describe the abundance of the relevant cell populations within post-sort fractions, providing details on the purity of the samples and how it was determined.*
Gating strategy	Cell populations are defined in the relevant figures and legends. Gating strategy provided in supplementary figures and described in the methods section under the title Flow Cytometry Analysis

☒ Tick this box to confirm that a figure exemplifying the gating strategy is provided in the Supplementary Information.
